# Role of digital health insurance management systems in scaling health insurance coverage in low- and Middle-Income Countries: A case study from Nigeria

**DOI:** 10.3389/fdgth.2022.1008458

**Published:** 2022-09-20

**Authors:** Okey Okuzu, Ross Malaga, Kenneth Okereafor, Ujulu Amos, Afolabi Dosunmu, Abiodun Oyeneyin, Victor Adeoye, Mohammed Nasir Sambo, Bassey Ebenso

**Affiliations:** ^1^ Instrat Global Health Solutions, Abuja, Nigeria; ^2^ School of Business, Montclair State University, Montclair, NJ, United States; ^3^ Department of Information / Communications Technology, National Health Insurance Authority, Abuja, Nigeria; ^4^ Adamawa State Contributory Health Management Agency, Yola, Adamawa State, Nigeria; ^5^ Ogun State Contributory Health Insurance Agency, Abeokuta, Ogun State, Nigeria; ^6^ Ondo State Contributory Health Commission, Akure, Ondo State, Nigeria; ^7^ Leeds Institute for Health Sciences, University of Leeds, Leeds, United Kingdom

**Keywords:** scale-up, digital technologies, health insurance, universal health coverage, Nigeria

## Abstract

**Background:**

Increasing global commitment to Universal Health Coverage (UHC) in the past decade has triggered UHC-inspired reforms and investments to expand health service coverage in many Low- and Middle-Income Countries (LMICs). UHC aims to ensure that all people can access quality health services, safeguard them from public health risks and impoverishment from out-of-pocket payments for healthcare when household members are sick

**Aim:**

This paper reviews the role of health insurance as a policy tool to address health financing as a contributory mechanism for accelerating the achievement of UHC in LMICs. We focus on Nigeria's legal framework for health insurance coverage for its whole population and the role of technology in facilitating enrollment to health insurance schemes.

**Methods:**

From May to July 2022, we adopted a cross-sectional case study design combining: (i) a literature review of the effects of UHC with (ii) document analysis of health insurance systems in Nigeria, and (iii) secondary analysis of health insurance datasets to understand experiences of deploying MedStrat, a locally-developed digital health insurance management system, and its features that support the administration of health insurance schemes in multiple states of Nigeria. We drew on contemporary technology adoption models to triangulate diverse data analyzed from literature and documents reviews and from health insurance datasets to identify: (i) enablers of adoption of digital insurance schemes, (ii) the contribution of digital technology to expanding access to health insurance, and (iii) further scalability of digital insurance intervention.

**Results:**

Preliminary findings suggests that digital insurance management systems can help to increase the number of enrollees for insurance especially among poor households. Three contextual enablers of adoption of digital insurance schemes were a favourable policy environment, public-private-partnerships, and sustained stakeholder engagement and training.

**Discussion and conclusion:**

Key elements for successful scaling of digital health insurance schemes across Nigeria and similar contexts include: (i) ease of use, (ii) existing digital infrastructure to support electronic insurance systems, and (iii) trust manifested *via* data encryption, maintaining audit trails for all data, and in-built fraud prevention processes. Our findings affirm that digital health technology can play a role in the attainment of UHC in LMICs.

## Introduction and literature review

Increasing global commitment to Universal Health Coverage (UHC) in the past decade has triggered UHC-inspired reforms and investments to expand health services in many Low- and Middle- Income Countries (LMICs) (1). UHC means that all people irrespective of their living standards or ability-to-pay, have access to needed and high-quality health services without suffering catastrophic financial hardship from using health services (2). According to the World Bank, the goals of UHC are to ensure that all people can access quality health services, to safeguard them from public health risks, and to protect them from impoverishment due to illness, whether from out-of-pocket (OOP) payments for health care or loss of income when a household member falls sick (3). On the basis of equity of access to healthcare, UHC is defined as a combination of: (i) legal assurance of health insurance to all citizens and legal residents of a country, (ii) accessibility to insurance coverage for >90% of the population—also called universal health insurance—including previously uninsured groups, and (iii) access to skilled birth attendance for >80% of the population (4). On a global scale, Erlangga et al. (2019) conducted a systematic review of the literature to determine the impact of health insurance on health care utilization in LMICs (5). They reported, “*Overall, health insurance schemes in LMICs have been found to improve access to health care as measured by increased utilization of health care facilities (32 out of 40 studies)*.”

Additional to the above systematic review, country case studies have reported the effects of pursuing universal health insurance policies. For example, a Turkish study of the effects of universal health insurance for uninsured population groups (including low-income households and the unemployed), revealed that a non-contributory health insurance combined with a Green Card scheme (6) increased access to health care services for poor households. Similarly, an evaluation of a 13 districts' community-based health insurance scheme in Ethiopia that was eventually scaled-up nationwide (7) showed: (i) improved availability of drugs and other supplies at health facilities, (ii) improved quality of health services and, (iii) increased health service utilization.

In 2015, Member States of the United Nations adopted the 2030 Agenda for Sustainable Development and its accompanying Sustainable Development Goals (SDGs), with the third goal of the agenda (SDG-3) aspiring to ensure good health and well-being for all. Member states also identified the achievement of UHC as the core target for assessing progress towards SDG-3.

A recent multi-country cross-sectional study conducted by Ranabhat et al. (2018) using diverse data sources from 193 UN member countries (8) revealed that universal health insurance and UHC facilitated: (i) the provision of a broad range of health services including child vaccination and sanitation, and (ii) significantly improved life expectancy at birth and healthy life expectancy. Ranabhat and colleagues concluded that implementing context-relevant programmes to achieve UHC held promise for improving global health outcomes and for achieving sustainable development goals (SDGs).

Across the African region, a range of technical actions, investments, innovations and monitoring approaches for health systems development have been adopted by countries to accelerate achievement of UHC. While Ghana (9) and Ethiopia (7) have identified and are tackling the inhibitions imposed by their health policies, South Africa (10) has devoted portions of its current National Digital Health Strategy to pursue four strategic principles namely: (a) expanded access to services for effective health coverage, (b) innovation for sustainable digital health impact, (c) digital health workforce driving economic development, and (d) whole-of-government approach.

In December 2016, the World Health Organization (WHO) Regional Office for Africa developed a framework of actions for health systems strengthening towards UHC in the context of the SDGs in the region. The framework was later adopted by the 67th session of the Regional Committee for Africa in August 2017. According to a 2022 WHO assessment report titled*:* *Tracking Universal Health Coverage in the WHO African Region*, (11)*,* the effects of universal health insurance and UHC in the African region can be categorized into two broad areas namely exploration of service coverage and exploration of financial risk protection.

### Service coverage index

The objective of the service coverage index (SCI) of UHC is that people in need of essential health care services receive them, and that the services received are of sufficient quality to achieve potential health improvements. Over the past 20 years, all countries in the African region saw an increase in the UHC SCI, with nine countries exceeding the 25% benchmark. In general, the African region saw on average, an increase of 22 index points in health service coverage (from 24% in 2000 to 46% in 2019), which contributed to a gain of nine years of healthy life expectancy from 47.1 years to 56.1 years over the same period. High/upper middle-income countries had much higher UHC SCI and life expectancy at birth than lower-income countries. It is important to highlight although the sharp rise of nine years in life expectancy in the WHO African Region is greater than in any other region of the world during the 2000–2019 period, it is still well below the global average of 64 years. Over the same period, global healthy life expectancy increased by only five years.

### Financial risk protection

While the African Region had extended service coverage, no major increases in catastrophic health spending were seen over the past seven years (i.e., 2010–2017 for which data was available for countries in the region). The report (11) suggests that while SCI increased from a population-weighted average of 40 in 2010 to 44 in 2017, the share of the African population spending over 10% of their household budget on health OOP remained relatively unchanged (with a slight decline of 0.5 percentage points from 7.76% in 2012 to 7.26% in 2017). The foregoing findings suggest a wide scope for improvement in terms of: reducing financial hardship that people experience in the care-seeking process and ensuring financial risk protection across all populations (SDG indicator 3.8.2). The findings also suggest protecting vulnerable groups against the impoverishing effect of health payments should be a focus for African countries as they match towards the collective SDG goals of 2030

## Evolution of health insurance system in Nigeria

In the past two decades, the Federal Government of Nigeria (FGN) has implemented various financing mechanisms to stimulate utilization of services, improve the health status of citizens (12), and advance the adoption of digital health as a key enabler of UHC. For example, in June 2005, the FGN started the Formal Sector Social Health Insurance Programme (FSSHIP) of the then National Health Insurance Scheme (NHIS) aimed at: (i) reducing inequity in access to healthcare services, (ii) reducing financial hardships among service users, and (iii) expanding insurance coverage to at least 30% of Nigerians to expedite achievement of UHC by December 2015 (13). The NHIS started its coverage of citizens from the public sector with compulsory coverage of government workers later expanded to private sector employees (14–16).

An appraisal in 2015 identified five challenges of the NHIS. First, the non-mandatory nature of contribution limited the scheme's capacity to mobilize resources (17). Second, low enrollment (of 5%) arising from focusing first on federal public sector and private sector employees (14). Third, poor adherence and discontinuous use of NHIS by over 50% of enrollees. Fourth, lack of equity funds to cover vulnerable groups. Fifth, high out-of-pocket (OOP) spending by households. Nigeria's OOP expenditure, which is the highest in Africa (18), increased from 60% in 2000 to 77% in 2017 in contrast to Ghana's OOP spending that decreased by 10% over the same period (18). In response to these challenges, the NHIS was decentralized in 2016 (17) and an equity fund called the Basic Healthcare Provision Fund (BHCPF) was introduced to meet the needs of vulnerable groups (19) and improve coverage and quality of service delivery. Decentralization also led to creation of States Social Health Insurance Agencies (SSHIA) in 35 of 36 states of Nigeria, to increase enrollment in health insurance at the sub-national level as a mechanism for providing financial protection for all groups and driving the UHC Agenda in Nigeria.

However, despite implementation of social health insurance schemes by many state governments, a number of gaps remained regarding (20): (a) the impact of health insurance schemes at sub-national level on universal enrollment and financial risk protection for enrollees; (b) retention in and utilization of health insurance schemes at the state level; (c) role of health insurance in promoting equitable access and use of healthcare services by vulnerable groups; and (d) role of digital technology in shaping enrollment, provision and use of healthcare services.

To address some of the above gaps, Nigeria's President Muhammadu Buhari signed the country's National Health Insurance Authority (NHIA) Bill 2022 into law on 19 May 2022 (21) to regulate and integrate ongoing health insurance schemes in Nigeria. The new law (the NHIA Act): (1) makes health insurance mandatory for all Nigerians and for legal residents in the country; (2) enforces a basic minimum package of health services for all Nigerians across diverse health insurance schemes operating in Nigeria; (3) empowers the NHIA to work with State Social Health Insurance Agencies (SSHIAs) to accredit primary and secondary health care facilities to provide health insurance coverage to all Nigerians, (4) improves private sector participation in the provision of healthcare services; and (5) align the country with the global push for using health insurance schemes as a tool to achieve universal access to quality and affordable healthcare, (6) empowers the NHIA to drive the use of digital technologies to accelerate operations and processes among stakeholders in the health insurance eco-system in Nigeria.

## Digital health technology and access to health insurance

The NHIA visibly recognizes the relevance of Information and Communications Technology (ICT) in driving health insurance across all stakeholders as vividly captured in Section 3 Clauses l, v and w; and Section 13 Clause 6 of the NHIA Act. Digital health is contributing to the attainment of UHC, especially in Africa where many countries have deployed various degrees of automation of their healthcare and health insurance processes. In recent times, Nigeria (22, 23), Ghana (24–26), Ethiopia (27–29), South Africa (30), Kenya (31), and many other African states have embarked on massive ICT projects (32) to deepen the administration of health insurance across the entire value chain. ICT has assisted LMICs to improve patient safety and reduce the costs of care, and accelarate the attainemnt of UHC (33).

Ogundeji et al. (16) identified the role of ICT expertise for optimizing Health Insurance Management Systems (HIMS) in the administration and management of health insurance schemes to include among others: (1) The ability of information technology expertise to identify, register and enroll members from both formal and informal employees (determining which informal sector workers are to be exempted from contributions and (2) The ability of information technology expertise to routinely process and manage claims and payments to providers used by beneficiaries.

Similarly, Shan et al. (34) pointed out that the three benefits of digital health technologies are that they: (1) can be rapidly adapted to changing contextual and condition/disease-related requirements, (2) can be scaled quickly, and (3) have the potential to increase healthcare access. Knowing this and responding to this new law in Nigeria, local technology firms have developed and adapted HIMS to comply with Nigeria's Contributory Health Insurance Management Laws.

Much of the research on mobile health (mHealth) systems in LMICs has been mainly in the areas of health worker training, health service delivery, disease tracking and in the management of chronic conditions such as mental health. Zayyad and Toycan (35), for example, studied the sustainable adoption of health information technologies in hospitals in Nigeria. Qureshi (36) described how Nigeria utilized ICTs such as mobile phones and global positioning systems (GPS) to contain the 2014 Ebola outbreak in West Africa. Guo and Li (37) explored the use of artificial intelligence technologies in rural areas of developing countries. Naslund et. al. (38) examined the use of “*digital technology for supporting non-specialist health workers in the delivery of mental health care*” in LMICs (including Nigeria).

Since 2020, the NHIA has embarked on a digital transformation initiative to transition its semi-manual operations into fully automated systems cutting across connectivity, biometric enrolment systems, beneficiary identification and card services, generation of credible healthcare industry analytics, as well as a unified stakeholder collaboration platform. However, despite the growing adoption of ICTs in driving health insurance systems in African and LMICs, none of the studies cited above focused on scaling health insurance systems technologies. To fill the gap in literature, our paper aims to report the experience of deploying MedStrat, a locally developed digital health insurance management system currently deployed in multiple states of Nigeria. A secondary objective is to explore the effect of digital technology in boosting enrollment to health insurance, and equitable access to and utilization of healthcare services by vulnerable groups. As expanding public health insurance is the gateway to increasing access to healthcare facilities and financial protection in many LMICs (5), this focus represents an important contribution to the literature.

Following the World Health Organization (39), this paper defines scale up as the embedding of digital health product(s) into each level of the health system (policy, practices, workflows, and daily lives of health workers) to improve output (scope, quality, efficiency), outcome (coverage, utilization) or impact (morbidity or mortality) rather than regrading digital interventions as standalone initiatives (40). In this respect, the integration of digital health insurance processes into policy, practices, and workflows of health workers in multiple states of Nigeria represents scale-up of digital technology.

## Context of mother and child healthcare in Nigeria

The country operates a three-level health system: primary, secondary, and tertiary, with healthcare provision being an overlapping responsibility of federal, state, and local governments (41). The Federal Ministry of Health (FMOH) is charged with designing health policies, delivery of tertiary healthcare services, and providing technical support to and regulates state and local government levels. On the other hand, State Ministries of Health (SMOH) provide secondary healthcare services, technical support for and regulation of primary healthcare (PHC) services. The Local governments deliver PHC services. Healthcare workers at the PHC level include medical doctors, nurse/midwives, laboratory technicians and community health extension workers. Although skilled antenatal care providers and birth attendants exist in most health facilities, the facilities lack essential drugs and basic tools and equipment for delivering high-quality mother and childcare services, which in turn affect infant and maternal mortality. Regardless of significant decreases in maternal and infant mortality in Nigeria since 2000 by 56 and 33% respectively, these health indices are terribly high at 512 deaths per 100,000 live births and 67 deaths per 1,000 live births in 2018 respectively (42, 43). There are inter-regional disparities in the levels of the indices with the northern region (represented by Adamawa State) having the highest maternal mortality ratio of 1,549 deaths per 100,000 live births in 2015 compared to other parts of Nigeria (44).

## Methods

### Study setting

The study was undertaken in three states of Nigeria (Ondo, Ogun and Adamawa) that commissioned a local technology company, InStrat Global Health Solutions (https://instratghs.com/) to develop and deploy a bespoke digital health insurance scheme (the MedStrat Health Insurance Management System) for help expand health insurance coverage particularly among poor and vulnerable populations of the three states.

### Study design

This was a cross-sectional study that adopted a case study approach, defined as an approach used to explore, describe or explain events or phenomena in their everyday contexts in order to generate an in-depth, multi-faceted understanding of a complex issue (45). Case studies are an established research design used extensively in a wide variety of disciplines, particularly in the social sciences. Often referred to as a “naturalistic” design in contrast to an “experimental” design (RCTs) in which researchers seek to control and manipulate variables of interest.

Stake (46) categorized three types of case studies: intrinsic, instrumental and collective. An intrinsic case study is typically used to learn about a unique phenomenon while the instrumental case study uses a particular case (some of which may be better than others) to gain a broader appreciation of an issue or phenomenon. The collective case study involves examination of multiple cases concurrently or serially, allowing for comparisons across selected cases in order to generate a broader appreciation of a particular issue.

This paper employed a collective case study design to report the adoption of digital health insurance schemes State Governments in Ondo, Ogun and Adamawa states of Nigeria. Additionally, the paper aims to understand the contextual enablers of adoption of digital health insurance and the influence of digital technology on the enrollment of pregnant women to health insurance, and their use of mother and child health (MCH) services.

Between May and July 2022, data collection for this cross-sectional study combined: (i) literature review of the effects of UHC—see the introductory section of this paper, with (ii) documents review of the evolution of health insurance systems in Nigeria, and (iii) secondary analysis of anonymized health insurance datasets obtained from the National and State Social Health Insurance Authorities. As this paper aimed to understand how digital technology shaped the adoption of health insurance by state governments, and enrollment of pregnant women for insurance in the three states, the documents reviewed as part of data collection for the study included: (i) state MCH policies, (ii) ICT policies, and (iii) reports of MCH programs to understand how the digital health insurance schemes were meant to work. The documents reviewed by the authors of this paper were used both as evidence for study findings (e.g., quantitative data extracted insurance datasets) and as an explanation for the study findings (e.g., data from state ICT policies).

## Conceptual framework: technology adoption models

Labrique et al. (47) identified five main success factors for scaling digital health solutions in LMICs. These are that: (1) the initiative must offer tangible benefits that address an unmet need and have user input, (2) stakeholders need to be, “*engaged, trained and motivated to implement a new initiative*”, (3) the technology used needs to be simple and adaptable, (4) there should be alignment with broad policy goals to ensure sustainable funding, and (5) the overall ecosystem needs to enable the initiative (e.g., digital infrastructure). We used the above factors to develop a framework for building trust among users of digital technology (see Table 2).

The findings of Labrique et al. (47) are bolstered by long standing frameworks for technology adoption. For example, the Technology Adoption Model (TAM) (48) suggests that when users are presented with a new technology its perceived usefulness and perceived ease-of-use will determine whether the technology will be adopted. For instance, a person's motivation to use an emerging technology in the context of healthcare is believed to be higher if that technology is easy to use during healthcare delivery. The classic TAM has been extended numerous times. For instance, the modified Technology Acceptance Model (mTAM) (49) also called TAM2 (50) studied and extended the classic TAM by acknowledging the influence of contextual factors in shaping technology acceptance in an organizational context and added a number of new factors to the model, such as but not limited to job relevance and the quality of the system output. Specifically, Ebenso et al.'s (51) adoption of TAM2 as part of the evaluation of a mother and child health care programme in Nigeria facilitated the identification of six organizational determinants of technology acceptance: (1) prior training to prepare frontline health workers to embrace technology, (2) access to technology in health facilities, (3) technical support for users of technology throughout the lifetime of a digital health project, (4) workload following technology introduction, (5) access to internet connection, and (6) regular electricity supply for accessing digital platforms and (re-)charging of digital devices. Moreover, TAM3 (52) further extended the classic TAM by including trust as an additional factor in the context of e-commerce systems. As HIMS deals with very private data of service users, we included this factor in our framework outlined in Table 2. Classic TAM and its extensions have been used in various research contexts. The most relevant is the study by Rahimi et al. (53) in which their systematic review of the literature found 134 papers that used TAM (or extensions) in a health care setting.

## Digital health intervention (MedStrat health insurance management system)

To address inequitable access to MCH services in western Nigeria, and to accelerate achievement of UHC, in 2019, a local technology company, InStrat Global Health Solutions (https://instratghs.com/) developed a digital insurance platform - the MedStrat Health Insurance System -, as a fully home-grown Health Insurance Management System (HIMS). This initiative was in response to a call by the Ondo State Ministry of Health, with whom InStrat had collaborated with for several years, for an HIMS that would digitize health insurance management activities and comply with Nigeria's Contributory Health Insurance Management Laws. The MedStrat HIMS fully automates all health insurance processes and supports paper-based back up where internet connectivity is insufficient or unavailable. The functionalities include electronic beneficiary enrollment, *via* a mobile application or the web portal management, health facility enrollment and management, electronic claims submission and processing; and real time reporting. MedStrat HIMS incorporates multi-step approvals for most processes to increase integrity and oversight of approval decisions. Approved services and drug lists and prices are maintained for all services including those for which prior authorizations are necessary. MedStrat integrates with all 3rd party payment gateways to process premiums electronically for Plan Types requiring premiums.

MedStrat HIMS is built around the following eight pillars:

1. Beneficiary Enrolment and Management

This service is accessible *via* an Android Mobile Application that is downloadable from Google Play Store and *via* the MedStrat HIMS Beneficiary Portal. The Beneficiary Portal includes the Beneficiaries' selected health plan, their health facility visit history, their profile and an image of their Beneficiary ID Card. The Mobile Application allows the Agents of the State Health Insurance Agency or Third-Party Organizations to enroll beneficiaries, capturing all their required information including photographs and completing their registrations. If registrations are completed offline, the system synchronizes with the secure remote cloud servers to upload newly registered beneficiaries to the system.

2. Provider Management

Provider Management is conducted through a web portal that contains information about activities and services of all authorized providers of medical services in each of the States and are expected to be assigned beneficiaries. Services provided and funded by the State Health Insurance (SHI) schemes are contained in the system including medical, dental and laboratory services. Price lists are maintained for all services and medications covered by the SHI.

The Provider Portal contains information about its current list of enrolled beneficiaries; patient encounter documentation; claims management processing, prior authorization request management; referrals management and reports and analytics.

3. Claims Management

A high integrity claims management process is critical to the success of health insurance systems. The MedStrat HIMS system allows healthcare providers to complete Individual Claims Form (ICF) for patients after every encounter and submit to the Provider Portal for every Patient. At the end of the month, the HIMS system consolidates information from all ICFs submitted and automatically populate the Provider Claims Form (PCF). The healthcare Provider may also provide backup information that will support individual claims. The claim is reviewed through a multi-level process supported by an electronic workflow that allows for defined roles, system access levels and controls.

4. System Administration

The HIMS is administered *via* the Administration Portal which allows the following functions to be seamlessly performed by the SSHIA staff: Beneficiary Management; Provider Management; Referral Management; Staff Roles and Access Management; Third Party Agent Management; Management of Approved Services and prices including Laboratory and Medicine. The Administration Portal also maintains a real-time Activity Log that allows review or audits of all actions taken on the HIS.

5. Customer Communications

MedStrat HIMS allows direct communication with clients and providers as follows:

**Beneficiaries:** Outbound SMS messages upon enrollment confirmation, plan renewal or changes to assigned facility. Bulk SMS messaging is supported to all beneficiaries or different beneficiary groups (e.g., by Plan Type).
**Healthcare Providers:** Outbound SMS and email upon Provider enrollment confirmation; initiation/receipt of referral codes; claims approval, query or denial, initiation/receipt of prior authorization codes. Bulk SMS and emails can also be sent to all Providers or different groups (e.g., by Local Government Area or Ward).

6. Reporting, Analytics and Data Visualization

While MedStrat HIMS maintains a reporting and data access portal, it is also linked to a robust 3rd Party Business Analytics web portal allowing visually appealing and user-friendly representation of real-time HIMS data including beneficiary enrollment details and statistics; Provider details, Claims and Financial information on Submitted Claims and Capitation Payments due.

7. Training and Technical Support

InStrat provides direct training and hands-on support to State Health Insurance Agency and third party agents. Supplementary training is also provided *via* online tutorials (available on InStrat's VTR Mobile Training Application), *via* a detailed instructions manual developed for each client (i.e., State Health Insurance Agency) and *via* cheat sheets developed for important HIMS systems processes.

8. Fraud and Abuse Prevention

InStrat has built important fraud prevention technologies and processes into the HIMS to mitigate risk. At the system level the HIMS maintains a full audit-trail of all activities by anyone with access to the system and all data is encrypted while in transit, under processing or in storage.

In addition, the HIMS provides the following:

• The beneficiary ID format contains an important algorithm that prevents forgery and can be detected easily by any Provider or Agency staff.
• All Individual Claims Forms can only be for patients whose coverage it verifies by their valid beneficiary enrollment number as patient information fields such as name, date of birth etc. are autofilled based on Beneficiary policy Number.
• The covered services based on Plan type is autofilled based on Beneficiary policy Number and set to a maximum number of services. Submission of claims for services not on the approved list will be accepted by the system only if the Prior Authorization Approval Code is provided.
• The MedStrat HIS will only accept Provider Claims Form (PCF) from authenticated providers.

While the above eight pillars represent standard MedStrat HIMS functions, InStrat has collaborated with its clients to customize them to suit their schemes' requirements. Feature sets are customized for the numbers of, and differences in plan types, benefits, and administration of those benefits, claims adjudication and approval processes, and beneficiary and provider communications. Unique features are also developed to address unique use cases.

## Results

### MedStrat HIMS clients

**Figure 1:**
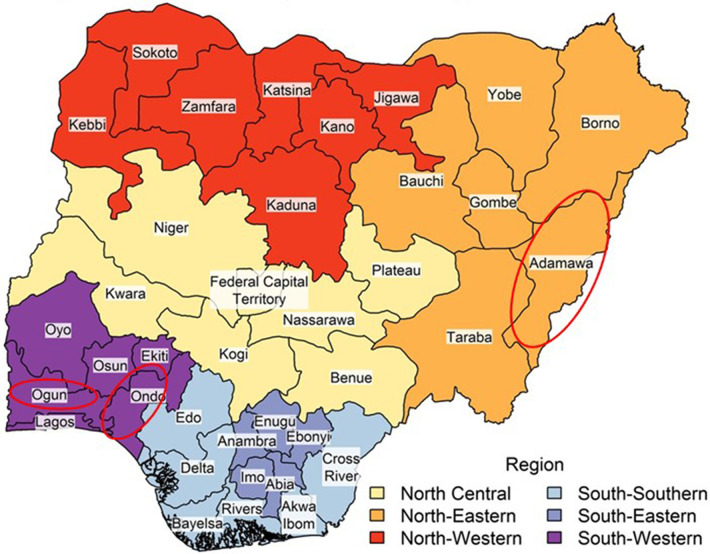
Map of Nigeria showing the states of Nigeria and the federal capital territory.

The MedStrat HIMS is currently implemented in the following three states of Nigeria: Adamawa in the Northeast, and in Ogun and Ondo states in the southwest of Nigeria (see red circles in Figure 1 for locations of the states). These states selected MedStrat after competitive bidding by several local and international health insurance system providers.

### Ondo state contributory health insurance commission: https://odchc.on.gov.ng/

On 6 February 2018, the State Governor signed into law, a bill to establish the Ondo State Contributory Health Commission (ODCHC) to provide health insurance for all residents of the state. As mentioned earlier, the State Government commissioned InStrat to develop the MedSrat HIMS, which it adopted in December 2019 for administering health insurance to its population, currently estimated to be 4.7 million (54).

Between 2019 and June 2022, a total of 37,946 people have benefitted from health insurance, 20,557 of which (or 54.2%) are pregnant women and children under age 5 funded through the State's equity fund named the Abiyamo Maternal / Child Health Insurance Scheme (AMCHIS), implemented at seven public hospitals in the state (55). See Table 1 for details. Preliminary analysis suggest that the numbers of people insured has evolved with the Ondo State's digital health insurance management system facilitating enrolment of previously uninsured groups and households that include pregnant women and children. Our analysis also indicated that over 6,689 pregnant women among the 20,557 enrollees have delivered their babies by December 2021, suggesting that maternal mortality among health insurance beneficiaries is 142/100,000 live births at the end of December 2021 compared to the National index of 512 maternal deaths/100,000 live births in 2018. While this is promising, we will continue to track maternal morbidity and mortality indices among insurance and non-insurance beneficiaries in Ondo State to better understand the contribution(s) of the digital health insurance management system and other contextual factors on mortality rates over time. MedStrat HIMS system was subsequently scaled up to Ogun and Adamawa states following 15 months of successful deployment in Ondo State.

**Table 1:** State client, beneficiary, and provider enrollment into MedStrat HIMS.

State Insurance Agencies that adopted MedStrat System	MedStrat adoption Date	Number of Health Insurance Plans	Beneficiaries Enrolled to date
ASCHMA	23/09/2021	7	Total: 31,809 № funded *via* equity fund: 31,020 (or 97.5% of enrollees)
OGSHIA	26/05/2021	5	Total: 49,497 № funded *via* equity fund: 47,327 (or 95.6% enrollees)
ODCHC	03/12/2019	5	Total: 37,946 № funded *via* equity fund: 20,557 (or 54.2% enrollees)

### Ogun State Health Insurance Agency (https://ogshia.org/)

The Ogun State Health Insurance Agency (OGSHIA) was created on 27th of May 2019 after the successful passage of the Health Insurance Bill by the Ogun State House of Assembly which the state Governor signed into Law No: 039/2018. The Health Insurance Agency then adopted the MedSrat HMIS in May 2021 for administering health insurance for Ogun State's estimated population of 5.2 million (54). Since adopting digital HIMS in May 2021, a total of 49,497 beneficiaries across 20 LGAs of Ogun state have been enrolled in the health insurance scheme, 47,327 enrollees (of 95.6%) who are pregnant women and children funded through Ogun State equity fund.

### Adamawa State Contributory Health Insurance Management Agency (https://aschma.org/)

The Adamawa State Contributory Health Management Agency (ASCHMA) was established by the Adamawa State Law No:18 of 2018 to provide quality and affordable health care services to all citizens of Adamawa State whose population is estimated to be 4.3 million (54). The state then adopted digital HIMS in September 2021. A total of 31,809 beneficiaries across 12 LGAs of the state have so far been enrolled for the health insurance scheme, which is implemented in partnership with the National Health Insurance Scheme (NHIS). 31,020 enrollees (or 97.5%) of 31, 809 beneficiaries are pregnant women and children funded through the Adamawa State equity fund.

### Scaling MedStrat HIMS for accelerating the achievement of UHC

Being a novel use of technology to support a relatively new health insurance scheme, InStrat placed significant emphasis on building the capacity of all the State health insurance agencies to increase their understanding of the functions of the HIMS system and how to interact with it. The three levels of the training process were as follows:

#### First level training

(1) The heads of the State Social Health Insurance Agencies and their core Agency ICT Staff were trained on all modules contained on the HIMS. This training was designed to allow the ICT staff to carry on the technical operation of the system. (2) All other departments that played roles in the health insurance scheme such as Accounts, Claims Processing, Human Resources were provided hands-on training on parts of the HIMS that affect their job functions.

#### Second level training

InStrat collaborated with core Agency staff to train healthcare providers, Third Party Administrators and Local Government Area focal persons on the modules of the health insurance system that were relevant to them.

#### Third level training and post-deployment supervisory support

Designated InStrat technical officers and State Social Health Insurance Agency ICT staff conducted joint field visits to provide on-the-job training and monitoring support to health care providers and Third-Party Administrators (TPA) for up to three months after the initial training provided to ICT staff. After this, full responsibility for further training and ongoing support is transitioned to state insurance agency ICT personnel.

InStrat also provided all MedStrat users with training material that are deployed *via* its proprietary Video Training (VTR) Mobile platforms for training frontline health workers. The VTR mobile training platform has been reported elsewhere (40). The materials used for the three levels of HIMS trainings included instructional videos, a full training manual and cheat sheets on important operational aspects of the system.

### A proposed framework for scaling HIMS

If we align Labrique, TAM, TAM2 and TAM3, we define a new framework for scaling HIMS in LMICs as follows:

1. The initiative must be supported by policy to ensure continued funding (Labrique)
2. Stakeholders must be “*engaged, trained, motivated”* (Labrique, TAM2)
3. The HIMS must be useful to its users (Labrique, TAM)
4. The HIMS must be easy to use (Labrique, TAM)
5. The digital infrastructure needs to be in place to support the HIMS (Labrique)
6. The HIMS needs to include elements that will build trust with users (TAM3)

## Discussion

The application of ICTs has shown great potentials for accelerating health insurance in Nigeria, as evidenced by:

1. the provisions for digital transformation in relevant portions of the new NHIA law,
2. adoption of functional HIMS platform by SSHIAs in Nigeria,
3. increased digital technology awareness and acceptance within the health insurance eco-system,
4. yearning for increased funding for health insurance digitization projects in Nigeria and other LMICs.

Social health insurance in Nigeria is mostly financed through the Basic Healthcare Provision Fund from the Federal Government Annual Grant of not less than 1% of its consolidated revenue fund, as well as grants from international donor partners. State Governments have supplemented this funding with their own resources, especially for vulnerable populations (56, 57). For example, Ondo State Government has dedicated approximately 1% of its revenues to fund health insurance for vulnerable populations. Low State Government revenues and competing social priorities means that health insurance funding is not always provided in Nigeria, let alone funding for specific aspects especially digital health. By 31 December 2021, Ondo State had provided health insurance coverage for some 37,946 beneficiaries across 18 Local Government Areas through the Basic Healthcare Provision Fund Programme. 20,557 (or 54.2% enrolees) of the 37,946 beneficiaries were vulnerable pregnant women and children covered through the Abiyamo Mother and Child Health Insurance Scheme (AMCHIS) of the Ondo State Government. Over time, a range of different MCH programmes implemented in Ondo State coupled with the ongoing digital health insurance coverage have allowed Ondo State to achieve a 78% reduction of maternal mortality rate from 775 maternal deaths per 100,000 births in 1998 to 170 maternal deaths per 100,000 live births in 2018 including but not sorely through its Abiye “*incentives*” program and the “*Agbebiye*” safe motherhood program that enabled Traditional Birth Attendants (TBAs) to transfer women who would have wanted to give birth at home to formal hospital settings to deliver their babies (42, 58).

It is important to highlight that the Abiye MCH initiative combined the above Abiye “incentives” and the “*Agbebiye*” components into a comprehensive maternal health program, developed by the Ondo State Government in February 2010 in response to a 2008 National Demographic and Health Survey report, which showed that Ondo State had the worst maternal outcomes in Southwestern Nigeria following years of under-funding, inadequately equipping, and poorly staffing health facilities (59). The Abiye MCH initiative, piloted in only two healthcare facilities in two LGAs of Ondo State, aimed to address the four key phases of delay in access to maternal health care services. These are: (1) delay on the part of pregnant women to seek care when experience complications, (2) delay in reaching healthcare due to poor infrastructure support, communication challenges and transport, (3) delay in accessing care due to poor facilities or absence of health facilities, and (4) delay on the part of the health system to refer “at risk cases or emergencies” (60). The “incentives” component of the Abiye MCH initiative worked with local communities to register all pregnant women in the two LGAs and to provide maternity services free-of-cost to pregnant women to boost demand for maternity services. Conversely, the Agbebiye safe motherhood component partnered with TBAs to transfer pregnant women managed by them to formal healthcare providers facilitated by providing TBAs with mobile phones to facilitate supportive supervision of the TBAs. But despite the comprehensiveness of the programme, the implementation of the Abiye MCH initiative in only two of 18 LGAs in Ondo State inadvertently limited access to MCH services for pregnant women in the remaining 16 LGAs of the Ondo State. To address the inequitable access to MCH services and promote sustainable financing for MCH services, the Government of Ondo State, in December 2019, designed a new health insurance scheme called the Abiyamo Mother and Child Health Insurance Scheme (AMCHIS) currently implemented in eight secondary health facilities where the Government pays insurance premiums on behalf of pregnant women and children under the age of five years. Through an agreed bundled tariff at the eight selected secondary health facilities across three senatorial zones, pregnant women from all 18 LGAs of Ondo state can access MCH services free-of-cost using AMCHIS.

Our results showed that in Ondo state, just over 50% of health insurance enrolees (pregnant women and children) were funded *via* the Ondo state equity fund with plans for incremental expansion of the equity funded to cover over 450,000 beneficiaries over a four-year period, including pregnant women, disabled persons and the general population (61). The equity fund forms an aspect of the Abiyamo MCH insurance scheme. Similarly, preliminary results from Ogun state showed that nearly 96% of 49,497 of health insurance enrollees are pregnant women and children funded through Ogun state equity fund. And 98% of 31, 809 health insurance beneficiaries in Adamawa state were pregnant women and children funded through the State equity fund.

The COVID-19 pandemic exacerbated the poor healthcare funding situation by compelling State Governments to redirect aspects of their health budgets to COVID-19 interventional efforts including setting up quarantine centers, COVID testing, sanitization zones at the expense of ICT-related investments. International donor partners have typically preferred to fund interventions for disease specific programs such as Malaria, HIV/AIDS and Tuberculosis. These disease-oriented funding mechanisms do not fully integrate digitization into their programing and they fail to recognize the umbrella role that health insurance schemes play in facilitating health care delivery and interventions across disease matrices.

### Lessons learned from MedStrat HIMS for scaling up digital health insurance schemes

Implementing MedStrat HIMS across multiple states of Nigeria underlined three wider contextual enablers of scale-up of digital insurance schemes namely favourable policy environment, public-private-partnerships, and sustained stakeholder engagement.

a) **Favourable policy environment:** Our analysis showed importance of the National Health Insurance Authority Act for obligatory nationwide adoption of digital health technology as a priority strategy for achieving the goals of increasing health insurance coverage and of accelerating the attainment of UHC (51). Similarly, favourable policy contexts in Adamawa, Ogun and Ondo States facilitated the adoption of MedStrat HIMS mainly because the objectives of the MedStrat project were aligned to the Federal Government's commitment as well as the global push for using health insurance schemes as a tool for broadening access to quality and affordable healthcare especially vulnerable mothers and infants.
b) **Public private partnerships:** The NHIA Act also promoted public-private-partnerships (PPP) in this case, between the national, state governments and technology companies for co-designing and deploying digital health insurance schemes for public benefit. Since 2013 when the NHIA embarked on aggressive fortification of the capacity of its ICT workforce, there have been no fewer than eighty (80) vendor proposals presented to the NHIA for digital health insurance implementations at different levels. While many of the digital proposals were general purpose health insurance computerized systems, others were specific to core health insurance operations particularly eClaims modules, patient encounter management systems, emergency services gateway, mobile health solutions for beneficiary access, enrolment systems, health insurance payment systems, health analytics tools, connectivity solutions optimized for health insurance stakeholders, as well as digital solutions for automating processes in Health Maintenance Organizations (HMOs).

It is interesting to note that, of all vendors that ever proposed digital solutions to the NHIA, about 80% of vendors who submitted proposals to the NHIA usually claim to have existing national spread or possess the technical potential to offer nationwide insurance coverage devoid of geographic limitations. While these claims are largely unsubstantiated, it however portrays the enthusiasm of technology service providers that target health insurance with the goal to advance UHC.

While the innovations offered by private service providers are commendable, the number and capability of these digital health insurance systems have continued to increase due to many reasons, including: (a) the growth in the stakeholder size in the Nigerian health insurance sector, (b) the influence of successful implementations such as the InStrat digital health solutions, (c) increased digital awareness due to COVID disruptions, as well as (d) the quest to use digital systems to foster compliance with emerging domestic and international regulations including the Health Insurance Portability and Accountability Act (HIPAA), the General Data Protection Regulation (GDPR), the Nigeria Data Protection Regulation (NDPR), NHIA Act, etc.

The PPP approach adopted in Nigeria boosted the feasibility of success for sharing resources, capabilities and project risks. In this case, InStrat Global Health Ltd provided the technological capability and digital health solutions while the Federal and State Governments provided healthcare infrastructure, ICT personnel for State Health Insurance Agencies and the population reach for ensuring successful digital health insurance projects.

c) **Sustained stakeholder engagement**: Table 2 highlighted the centrality of continuously engaging stakeholders (including the National Health Insurance Authority, State Social Health Insurance Agencies and their ICT personnel) though multi-level and on-the-job trainings, supportive supervision and feedback processes that ensured improvement of digital insurance processes.

**Table 2:** Elements for successful scaling of digital health insurance schemes in LMICs.

Elements of the framework	MedStrat HIMS
Policy support	• National health insurance law of 2022 • State-level insurance policies
Stakeholder engagement	• Provides prior training and technical support through the HIMS • Continuous engagement using community feedback to improve HIMS
Usefulness	Covers all functions for both beneficiaries and providers
Ease of use	• User friendly mobile app (Android) • Use of SMS for communication with beneficiaries and providers • Dynamic fields that are based on user roll
Infrastructure	• Backup (paper based) systems for areas with no or low connectivity • ID card contains QR code for off-line verification of beneficiaries • Bulk upload feature to handle large numbers of beneficiaries and providers
Trust	• All data is encrypted • Full audit trail for all data • Built-in fraud and abuse prevention

We have demonstrated that the role of digital health insurance management systems in scaling health insurance coverage in LMICs cannot be relegated. The success stories amplified in our multistate case study corroborate the near-indispensability of ICT in the operationalization of health insurance in LMICs and Nigeria. A fully automated, well-functional health insurance management system underpins health insurance schemes’ effectiveness in channeling resources for health care delivery at scale, especially for poor and vulnerable populations. By utilizing findings from Labrique, TAM and TAM3 success factors for the adoption of technology, we developed a new framework for analyzing the scalability of HIMS. With substantial backing from the new NHIA law which made clear provisions for the mandatory application of ICT in the health insurance eco-system, we applied this new framework to LIMCs - specifically Nigeria in this instance. We conclude that the current MedStrat HIMS checks all of the boxes in the framework and therefore can easily scale. Therefore, if more funds are made available (62) to SSHIAs, they could potentially provide health insurance coverage to significantly larger numbers of people thereby progressing towards Universal Health Coverage. Wider health insurance coverage translates to better health outcomes, which will also justify further investments in digital technology to sustain the upward trajectory of health insurance coverage growths and accelerate the achievement of several United Nations Sustainable Development Goals (SDGs) (63). Notable for health insurance enrollees in Nigeria include **Goal 1** of ending poverty through reducing catastrophic household expenditure; **Goal 3** of ensuring healthy lives and improving well-being though better health outcomes; and **Goal 5** of achieving gender equality through increased insurance coverage for pregnant women; and **Goal 10** of reducing inequality within and between countries though increased coverage of vulnerable people using health equity funds.

### Limitations and future directions

This paper has a number of limitations. First, it focuses only on Nigeria. There is no way to predict whether our findings are generalizable to other countries or contexts. Given the vast differences in the health insurance systems among countries, it is in fact likely that results elsewhere would differ. Research from other countries would provide valuable comparisons that would enhance the literature in this area.

Second, given the stages of implementation of digital health insurance schemes in the three states, this case study involved the review of literature and insurance databases. It did not involve empirical data collection/analysis, nor did it interview programme stakeholders for their views. Interviewing or surveying stakeholders is an obvious avenue for future research.

Third, the criterion for evaluation of positive impact of digital health interventions is based on a single vendor model. While this approach provides a credible basis for extrapolation from the three use case states, it also creates an opportunity for future adoption of a multi-vendor approach that can potentially confer stronger legitimacy to the claims, based on equity.

## Data Availability

The original contributions presented in the study are included in the article, further inquiries can be directed to the corresponding author/s.
